# Cord Blood Platelet Gel as a Treatment of Occipital Pressure Injuries in Newborns: Report of Two Cases

**DOI:** 10.3390/children8121079

**Published:** 2021-11-23

**Authors:** Silvia Ferrario, Alessia Zorz, Gabriele Sorrentino, Stefania Villa, Riccardo Cavalli, Fabio Mosca, Laura Plevani, Stefano Ghirardello

**Affiliations:** 1 Neonatal Intensive Care Unit, Foundation IRCCS Ca’ Granda Ospedale Maggiore Policlinico, Via della Commenda 12, 20122 Milan, Italy; silvia.ferrario@policlinico.mi.it (S.F.); alessia.zorz@policlinico.mi.it (A.Z.); gabriele.sorrentino@policlinico.mi.it (G.S.); fabio.mosca@policlinico.mi.it (F.M.); laura.plevani@policlinico.mi.it (L.P.); 2 Department of Transfusion Medicine and Hematology, Foundation IRCCS Ca’ Granda Ospedale Maggiore Policlinico, Via Francesco Sforza 35, 20122 Milan, Italy; stefania.villa@policlinico.mi.it; 3 Pediatric Dermatology Unit, Foundation IRCCS Ca’ Granda Ospedale Maggiore Policlinico, Via Pace, 9, 20122 Milan, Italy; riccardo.cavalli@policlinico.mi.it; 4 Department of Clinical Sciences and Community Health, University of Milan, Via Festa del Perdono 7, 20122 Milan, Italy; 5 Neonatal Intensive Care Unit, Foundation IRCCS Policlinico San Matteo, Piazzale Golgi 11, 27100 Pavia, Italy

**Keywords:** cord blood platelet gel, pressure injuries, newborn, case report

## Abstract

Background: A Pressure Ulcer (PU) is a severe event and could create discomfort to newborns. In newborns, one of mostly stricken location by PU is occipital area. Recent studies have highlighted that Cord Blood Platelet Gel (CBPG) might be a better alternative compared to traditional treatment. We report two cases of occipital PU treated with CBPG. Case report: Two male infants showing occipital PU were treated with standard local treatment, but no improvement was observed. After parental informed consent was obtained, CBPG application on PU was performed every 48 h. In these two cases of PU, a fast improvement in healing was observed since the first application of CBPG. The PU healed resulted in a scar after 53 and 50 days (Case 1 and Case 2, respectively) from development. No complications or infections were reported. Conclusions: CBPG contains many angiogenetic and growth factors, these characteristics make it indicated in treating soft tissue injuries. It would seem to be safe and an effective treatment of neonatal PUs reducing the time of the healing and the hospitalization and the infectious risks. Further studies are needed to evaluate long term aesthetic and functional results of PU treated with CBPG.

## 1. Introduction

Pressure ulcers (PU) are rarely reported in the neonatal population, with an estimated incidence in infants admitted to Neonatal Intensive Care Units (NICU) between 3.7% and 21.6% and a prevalence of 23% [1].

Endotracheal tube, non-invasive ventilation, vascular catheter, immobilization, hypotension, hypoxemia, prolonged NICU hospitalization, low birth weight, and prematurity represent risk factors for PU [1,2,3].

Generally, PU can be subdivided in conventional, due to pressure over bones prominence, or device-related, produced by pressure of a diagnostic or therapeutic device [4]. 

Conventional PU require a longer time to develop than device-related PU and are more common in infants with higher birth weight [4]. Occiput is the area at higher risk for developing conventional PU; indeed, the head is proportionately larger and heavier than the body [5,6]. 

A PU is a severe event associated with pain, worse sleep quality, neurobehavioral development, increased length of hospitalization, and increased risk of infection and toxicity due to treatment product absorption [2]. PU prevention and treatment are not standardized for the neonatal population and mainly refer to adults’ experience. However, most of the available products to treat PU are contra-indicated in newborns because of the risk of absorption and toxicity. Therefore, only a few products are available for the treatment of neonatal PU [1].

In the last few years, platelet gel (PG) derived from peripheral blood (autologous or allogenic) has been proposed to treat PU, due to its properties. Indeed, PG can stimulate and accelerate wound healing, reduce bleeding, and promote tissue regeneration and angiogenesis. In addition, it might have an anti-inflammatory, analgesic, and anti-bacterial effect [7,8,9,10,11].

A platelet gel derived from cord blood (CBPG) was recently obtained and used in regenerative medicine [12,13,14].

Our group has previously described the positive experience with CBPG, used in infants affected by dystrophic recessive epidermolysis bullosa [15,16,17].

CBPG in use contains a mean platelet count of 1000, range 800 to 1200 × 10^9^/L. CBPG is rich in growth factors such as platelet-derived growth factor, transforming growth factor, basic fibroblast growth factor, and vascular endothelial growth factor [18]. 

We describe the case of two newborns with occipital PU treated with CBPG, after the failure of conventional treatment.

## 2. Case Presentations

### 2.1. Case 1

#### Patient Information

A male infant affected by left-side congenital diaphragmatic hernia and treated with Fetoscopic Tracheal Occlusion (FETO) was born in June 2019 at 32 + 0 weeks of gestational age by cesarean section, his birth weight was 1800 g. He was immediately intubated at birth and transferred to the NICU. On day 2, he underwent surgical repair of the diaphragmatic breech with a Gore-Tex patch positioning. He required 33 days of mechanical ventilation and 21 days of non-invasive respiratory support (nasal continuous positive air pressure and high flow nasal cannula). Dobutamine, dopamine, milrinone, noradrenaline, and nitric oxide were administered in the first days after birth to correct systemic hypotension and pulmonary hypertension. Morphine and midazolam were administered for sedo-analgesia. After 19 days, an occipital PU developed. 

**Figure 1:**
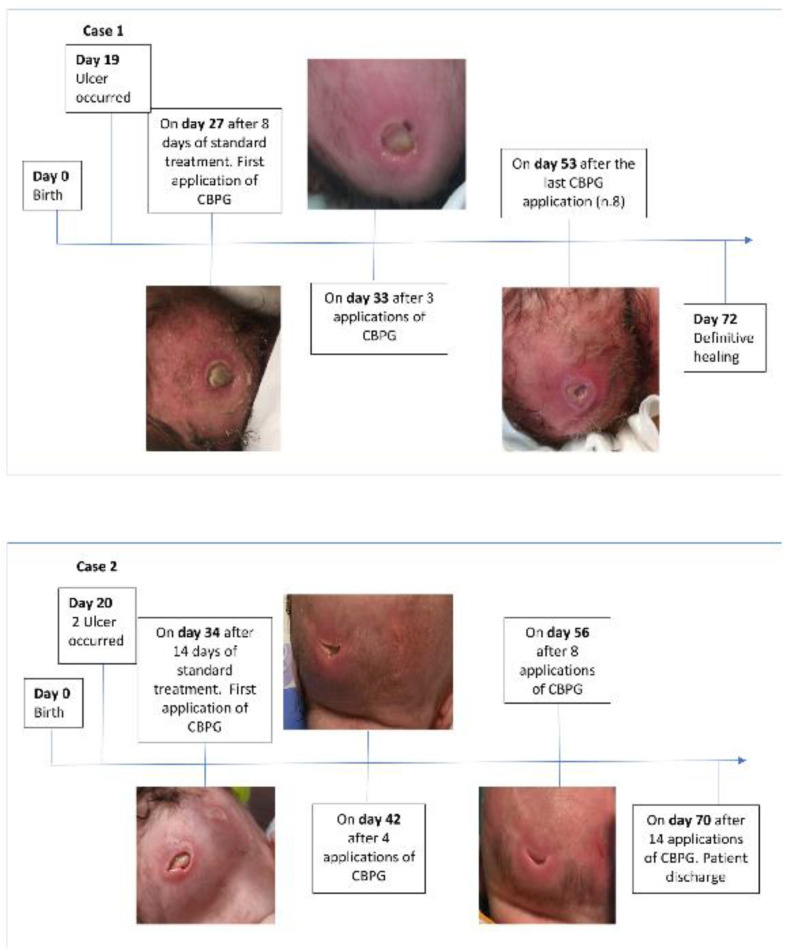
Case 1 and Case 2 timeline: from the development of PU to the definitive healing.

At that time, the ulcer was classified “unstageable” by National Pressure Injury Advisory Panel (NPIAP) scale because it was covered by eschar [19]. Standard local treatment with thin hydrocolloid dressing was performed for 8 days without benefits. Then, the ulcer was classified as stage IV of NPIAP and its size was 1.8 cm × 1.8 cm. On day 27 of life, after having obtained the parents informed consent form, CBPG was locally administered to improve tissue repair and the healing process. The area was cleaned with a sterile physiological solution, and CBPG was aseptically placed on the ulcer. CBPG was covered with a non-woven fabric gauze. CBPG was applied every 48 h; eight applications were necessary to significantly reduce the ulcer. Standard local treatment with hydrogel and thin hydrocolloid dressing was applied until complete resolution healing on day 72 (Figure 1).

### 2.2. Case 2

#### Patient Information

A male infant affected by a cervical region’s giant cystic lymphangioma was born in October 2020 at 38 + 6 weeks of gestational age by EXIT procedure. Birth-weight was 2740 g. He was immediately intubated and transferred to the NICU. On days 14 and 21, ultrasound-guided injections of sclerosing drugs (Doxycycline + Bleomycin) was performed to reduce the lesion, and administration of oral Sirolimus was started. On day 33, he was successfully extubated by video laryngoscopy procedure. On day 20, two occipital pressure ulcers developed due to deep sedation immobility and drug therapies.

Both ulcers were classified “unstageable” by NPIAP scale at the onset and were covered by eschar [19]. Standard local treatments with hydrogel and thin hydrocolloid dressing was started on the same day. No improvement was observed after 14 days of treatment, and both ulcers turned to stage IV of NPIAP; the sizes were 2 cm × 1.5 cm and 0.7 cm × 0.7 cm, respectively. After having achieved the parental informed consent form, on day 34 CBPG therapy was started. The portion of occiput was rinsed with sterile physiological solution and CBPG was applied on the ulcers using aseptic technique. CBPG was covered by an occlusive dressing of gauzes with paraffin and transparent film dressing. The smaller lesion was fully recovered after one application of CBPG. Fourteen applications were necessary to obtain final healing on day 70 (Figure 1).

### 2.3. CBPG Preparation

CBPG units were obtained after centrifugation of allogeneic cord blood units collected from placentas voluntarily donated to the Milano Cord Blood Bank by healthy mothers in term pregnancies, after informed consent, as previously described [20]. After sample collection, platelet gel was prepared by the centrifugation of cord blood unit. The bags containing the platelet concentrate were stored in a −80 °C freezer and when necessary for use, under aseptic conditions, the bags were defrosted in a warm water bath at 37 °C. The platelet concentrate was activated with commercial activator kit Plateltex^®^ ACT (Plateltex S.R.O., Prague, Czech Republic) following manufacturer’s instructions. After mixing the platelet concentrate aliquots with activator (9:1) by gentle agitation at 22 °C (±2 °C) for about 10 min, the gelation occurs spontaneously [21].

## 3. Discussion

PG obtained from adult peripheral blood is currently used in dental, orthopedic, ophthalmic and cardiac surgery, bone, muscle, tendon, skin repair, and diabetic foot ulcer treatment [7]. PG contains growth factors that promote tissue regeneration and have anti-inflammatory, anti-bacterial, analgesic, and hemostatic effects. PG obtained from discarded placentas (CBPG) has been used in pediatric settings in recent years [20].

CBPG shows immunomodulatory and tissue regenerative characteristics [22]. CBPG has a high level of viral safety; indeed, it is virtually Cytomegalovirus and Toxoplasma free [18].

Compared to that obtained from peripheral blood, CBPG contains different proteins, angiogenetic and growth factors, and hormones supporting fetal tissue formation; for these characteristics, CBPG is considered more effective in treating soft tissue injuries [12,18,22].

Hospitalized newborns should be carefully evaluated for the risk of developing PU and a complete inspection of the skin should be performed, paying attention to the high-risk areas. The occiput is one of the most common areas of neonatal PU. Immobility, sedation, hypotension, hypoxemia, mechanical ventilation, and extracorporeal membrane oxygenation are risk factors for occipital PU. Postural changes are recommended for occipital PU prevention and treatment every two hours, and therapeutic surfaces are suggested [1,23].

The treatment of neonatal PU is not standardized. There are fewer products available in the neonatal setting than in the adult population due to the increased risk of toxicity and adverse reactions. The neonatal population’s standard PU treatment is based on the reduction in local pressure and cleaning the affected area with a sterile physiological solution. The products to use should be chosen based on therapeutic goals: debridement, exudate or infection management, or healing stimulation. Available products are hydrogel, hydrocolloid, polyurethane foam, hydrofiber, silicone, silver, transparent semipermeable dressing [2].

In partial-thickness PU, the cutis regenerates, the damaged skin regains original characteristics due to the re-epithelialization process, epithelial cells migrate from borders to the middle of the lesion. Therefore, the deepest portion of the epidermis is characterized by big stem cells whose division replaces the lost cells in the most superficial portion of the cutis.

In full-thickness PU, the cutis does not regenerate but just repairs damage, therefore, newly formed tissue does not regain original characteristics. In fact, granulation tissue grows from the base of a wound and fills the ulcer, resulting in scar tissue without skin appendages, e.g., hair follicles, sebaceous, and sweat glands localized in derma [24].

Our local standard treatment for unstageable and IV stage PU consists of cleaning the lesion with a sterile physiological solution followed by local hydrogel application to fill the PU and hydrocolloid dressing for autolytic debridement.

Specifically, in the two patients described, we started standard treatment according to our local procedure, without significant improvement. Consequently we decided on CBPG application every 48 h. A fast improvement in PUs healing was observed since the first application of CBPG. The PU healed resulted in a scar after 53 and 50 days (Case 1 and Case 2, respectively) from development. In both cases, during CBPG treatment, no complications or infection were reported.

Recent evidence has suggested that treatment with CBPG could improve PU due to its potential effects reducing hospitalization time and care costs [14].

The antimicrobial activity of CBPG prevents the occurrence of infections creating a skin barrier, and, consequently, its complications. Moreover, studies have highlighted that CBPG has a hemostatic effect on the lesions and improves the healing process [15,16].

The healing time of neonatal PU is not defined, so we cannot compare our result with standard treatment. However, neonatal PU treatment with CBPG seems safe and might be an alternative, potentially more successful option for the treatment of neonatal PU.

To our knowledge, this is the first report of CBPG as treatment of neonatal PU.

Based on our observations, CBPG treatment could be the first-line treatment of neonatal PU, reducing the healing time and infectious risks.

Long-term aesthetic and functional results (skin elasticity, scar retractions, skin discolorations) should be evaluated over time and requires further studies.

## Data Availability

The data supporting the findings of this study are private due to patients’ privacy, but they are available from the corresponding author.
